# Zinc-Finger Protein 545 Inhibits Cell Proliferation as a Tumor Suppressor through Inducing Apoptosis and is Disrupted by Promoter Methylation in Breast Cancer

**DOI:** 10.1371/journal.pone.0110990

**Published:** 2014-10-31

**Authors:** Yun Xiao, Tingxiu Xiang, Xinrong Luo, Chunhong Li, Qianqian Li, Weiyan Peng, Lili Li, Shuman Li, Zhenyu Wang, Liping Tang, Guosheng Ren, Qian Tao

**Affiliations:** 1 Molecular Oncology and Epigenetics Laboratory, The First Affiliated Hospital of Chongqing Medical University, Chongqing, China; 2 Department of Endocrine and Breast Surgery, The First Affiliated Hospital of Chongqing Medical University, Chongqing, China; 3 Cancer Epigenetics Laboratory, Department of Clinical Oncology, State Key Laboratory of Oncology in South China, Sir YK Pao Center for Cancer and Li Ka Shing Institute of Health Sciences, The Chinese University of Hong Kong, Hong Kong, China; 4 Shenzhen Research Institute, The Chinese University of Hong Kong, Hong Kong, China; Roswell Park Cancer Institute, United States of America

## Abstract

Krüppel-associated box-containing zinc finger proteins (KRAP-ZFPs) are well recognized as key regulators of transcription, which play a crucial role in the regulation of cell proliferation, differentiation, apoptosis and tumorigenesis. We previously identified a KRAP-ZFP protein ZNF545 acting as a tumor suppressor involved in tumor pathogenesis. However, its expression and biological function in breast cancer remain elusive. In this study, we found that ZNF545 was frequently downregulated in estrogen receptor-positive (ER+), progesterone receptor-positive (PR+) and human epidermal growth factor receptor 2-negative (HER2−) breast tumor tissues compared with paired adjacent non-tumor tissues. We further examined its expression and methylation in breast cancer cell lines by semi-quantitative RT-PCR and methylation-specific PCR. We found that ZNF545 was silenced by promoter methylation in MCF7 cell line, and its expression could be restored by demethylation, concomitant with increased unmethylated alleles. ZNF545 methylation was detected in 29% of breast tumor tissues, but not in normal breast tissues, suggesting tumor-specific methylation of ZNF545 in breast cancer. Ectopic expression of ZNF545 in MCF7 cells inhibited cell proliferation through inducing cell cycle G0/G1 arrest and apoptosis, thus as a tumor suppressor. Moreover, ZNF545 upregulated mRNA and protein levels of c-Jun/AP1, BAX, p53 and Caspase 3. Taken together, these results demonstrate that ZNF545 inhibits breast tumor cell proliferation through inducing apoptosis and is disrupted by promoter methylation in breast cancer.

## Introduction

Breast cancer is one of the most common malignancies and the leading cause of cancer death in females worldwide, accounting for 23% (1,380,000) of all cancer cases and 14% (458,400) of cancer deaths in 2008 [1]. In the past 25 years, breast cancer death rates in many Western countries have been decreasing, mainly because of early detection through mammography and improved treatment. In contrast, in many Asian and African countries, both incidence and mortality rates have been rising [1]. Unfortunately, the detailed mechanisms causing breast cancer development are still unclear. Remarkably, epigenetic inactivation of tumor suppressor genes (TSGs) by aberrant promoter methylation has been proved to play a key role in the development of breast cancer [2]. Identification of more novel TSGs disrupted by promoter methylation would help to reveal molecular mechanisms related to the activation of tumorigenesis pathways and the inactivation of tumor suppressive pathways, facilitating the development of potential tumor markers for the early detection of breast cancer.

Zinc finger proteins (ZFPs), the largest transcription factor family, are only present in tetrapod vertebrate genomes [3]. About a third of ZFPs contain the Krüppel-associated box (KRAB-ZFPs) [3] and 51% of KRAB-ZFPs are clustered within a region close to 19q13 [4]. KRAB-ZFPs play a vital role in the regulation of multiple cellular progresses including cell proliferation, differentiation, apoptosis and tumorigenesis [3], [5], [6]. ZFPs act as transcription factors (TFs) by binding gene promoters to activate or repress gene expression [7], [8]. Several KRAB-ZFPs, such as ZNF382 and ZNF569, have been identified as tumor suppressors [9], [10], whereas others, such as ZNF217, GLI1, and ZNF147, serve as app:modword:serve asoncogenic proteins [7], [11], [12], [13]. However, the roles for most KRAB-ZFP family members in carcinogenesis remain ambiguous including breast cancer [14].

We previously demonstrated that Zinc-finger protein 545 (ZNF545) is a novel KRAB-ZFP member frequently methylated in multiple common tumors. Here, we examined expression and methylation of ZNF545 in breast cancer, and further assessed its tumor suppressive functions in breast cancer.

## Materials and Methods

### Cell lines and tumor samples

A series of breast tumor cell lines (BT549, MDA-MB-231, MDA-MB468, MCF-7, T47D, SK-BR-3) were used. These cells were from ATCC (American type culture collection). Cell lines were maintained in RPMI 1640 (Gibco BRL, Karlsruhe, Germany) supplemented with 10% fetal bovine serum (FBS; Invitrogen, Carlsbad, CA), and 100 U/ml of penicillin and streptomycin at 37°C in moist air including 5% CO2 [15], [16]. DNA and RNA samples of various primary breast tumor tissues, including paired surgical margin tissues and normal breast tissues, have been previously described [17], [18], [19]. Fresh cancer tissues and breast cancer adjacent tissues were obtained from patients who underwent primary surgery at the Surgery Department of the First Affiliated Hospital of Chongqing Medical University. ER and PR status has been determined by immunohistochemistry (IHC) assay, and HER2 was assessed through IHC and fluorescence in situ hybridization (FISH). Clinical and pathological data of all the participants were obtained, and their demographies are summarized in Table 1. This research was approved by the Institutional Ethics Committees of the First Affiliated Hospital of Chongqing Medical University (Approval notice: 2010/2012(23)). Our experiments were undertaken with the understanding and written consent of each subject, and the study methodologies conformed to the standards set by the Declaration of Helsinki.

**Table 1:** ZNF545 methylation and clinicopathologic features of breast tumors.

Clinicopathological features	Number (n = 128)	*ZNF545* promoter methylated status		*P* value
		methylated	unmethylated	
**Age**				0.232
≤40	14	2 (14%)	12 (86%)	
>40	102	33 (32%)	69 (68%)	
unknown	12	2 (17%)	10 (83%)	
**grade**				0.658
I	7	2 (29%)	5 (71%)	
II	81	26 (32%)	55 (68%)	
III	6	2 (33%)	4 (67%)	
unknown	34	7 (21%)	27 (79%)	
**Tumour size**				0.302
<2.0 cm	42	10 (24%)	32 (76%)	
≥2.0 cm≤5.0 cm	63	21 (33%)	42 (67%)	
>5.0 cm	9	4 (44%)	5 (56%)	
unknown	14	2 (14%)	12 (86%)	
**Lymph node metastasis**				0.371
Positive	54	18 (33%)	36 (67%)	
Negative	60	17 (28%)	43 (72%)	
unknown	14	2 (14%)	12 (86%)	
**ER status**				0.501
Positive	54	19 (35%)	35 (65%)	
Negative	43	12 (28%)	31 (72%)	
unknown	31	5 (16%)	6 (84%)	
**PR status**				0.403
Positive	44	14 (32%)	30 (68%)	
Negative	53	17 (32%)	36 (68%)	
unknown	31	6 (19%)	25 (81%)	
**HER2 status**				0.068
>+++	6	2 (33%)	4 (67%)	
++	50	21 (42%)	29 (58%)	
<+	39	8 (21%)	31 (79%)	
unknown	32	6 (19%)	26 (81%)	
**p53 expression**				0.540
Positive	37	13 (35%)	24 (65%)	
Negative	49	14 (29%)	35 (71%)	
unknown	42	10 (24%)	32 (76%)	
**phase**				0.762
1	35	10 (29%)	25 (71%)	
2	54	17 (31%)	37 (69%)	
3	25	8 (32%)	17 (68%)	
4	1	0 (0%)	1 (100%)	
unknown	13	2 (15%)	11 (85%)	

### DNA and RNA extraction

Genomic DNA was extracted from cell lines and tissues using DNAzol Reagent (Invitrogen, Rockville, MD, USA) and the QIAamp DNA Mini Kit (Qiagen, Hilden, Germany) according to the manufacturer's protocols. Total RNA was isolated from cell lines and tissues using TRI Reagent (Molecular Research Center, Cincinnati, OH). The integrity of DNA and RNA were detected by gel electrophoresis. Samples were stored at −80°Cuntil use.

### 5-aza-2′-deoxycytidine and trichostatin A treatment

As previously described [15], cell lines were treated with 10 µmol/L 5-aza-2- deoxycytidine (Aza) (Sigma-Aldrich, Steinheim, Germany) for 3 days and further treated with 100 nmol/L trichostatin A (TSA) (Sigma-Aldrich, Deisenheim, Germany) for an additional 24 h.

### Reverse transcriptase–polymerase chain reaction and real-time PCR

The reverse transcriptase–polymerase chain reaction (RT-PCR) was performed as previously described [20]. Samples were placed in a 12.5 µl reaction mixture containing 2.5 µl of cDNA. lyceraldehyde-3-phosphate dehydrogenase (GAPDH) was amplified as a control. The primer sequences are ZNF545-F: 5′-GAGCCTTGGAAAGTTGTGAG-3′, ZNF545-R: 5′-GGCATTTTCACACTACTGAAG-3′. RT-PCR was performed with 35 cycles for ZNF545 and 23 cycles for GAPDH using Go-Taq (Promega, Madison, WI). Real-time PCR was performed according to the manufacturer's protocol (HT7500 system, Applied Biosystems), with the expression level of ZNF545 in normal breast tissues set as baseline.

### Bisulfite treatment and methylation-specific PCR

Bisulfite modification of DNA and methylation-specific PCR (MSP) were performed as previously described [21], [22]. Bisulfite-treated DNA was amplified by MSP with the methylation-specific primer set ZNF545-m1: 5′-TTTTTTTTAGGTTTTGTCGCGTC-3′, ZNF545-m2: 5′-CTACTAAAAAAACCGAACGCG-3′ or the non-methylation-specific primer set ZNF545-u1: 5′-TTTTTTTTTAGGTTTTGTTGTGTT-3′, ZNF545-u2: 5′-CCAAACACACTCACAAAATACA-3′. MSP was performed for 40 cycles using AmpliTaq-Gold DNA Polymerase (Applied Biosystems, Foster City, CA) with an annealing temperature of 60°C and 58°C, respectively. Methylated and non-methylated human DNA were used as positive and negative controls, respectively. The PCR products were identified on a 2% agarose gel containing 100 bp DNA markers (MBI Fermentas, Vilnius, Lithuania).

### Colony formation assay

Monolayer culture was performed. MCF7 cells (2×10^5^ per well) were plated in six-well plates and transfected with pcDNA3.1(+)-Flag-ZNF545 plasmid or control vector (4 µg each) using Lipofectamine-2000 (Invitrogen, Carlsbad, CA). Forty-eight hours later, transfectants were collected, re-plated, and selected for two weeks in the presence of 100 µg/ml G418 in MCF7 cells. Surviving colonies were stained with Gentian Violet and visible colonies (≥50 cells) were counted. All the experiments were performed in triplicate wells three times.

### Cell proliferation assay

MCF7 cells were cultured in six-well plates at a density of 2×10^5^ cells/well and grown overnight. Cultures were then transiently transfected with pcDNA3.1(+)-Flag-ZNF545 plasmid or the control vector using Lipofectamine-2000 (Invitrogen, Carlsbad, CA). Forty-eight hours later, 2×10^3^ cells were replated in 96-well plates. After 24 h, 48 h, and 72 h, proliferation was measured using the Cell Counting Kit-8 (CCK-8, Beyotime, Shanghai, China) [23]. The experiment was repeated three times independently.

### Analysis of cell cycle and apoptosis

MCF7 cells were cultured in 6-well plates at a density of 2×10^5^ cells/well and grown overnight. Cultures were then transiently transfected with 4 µg pcDNA3.1(+)-Flag- ZNF545 plasmid or the control vector using Lipofectamine-2000 (Invitrogen, Carlsbad, CA) following the manufacturer's protocol. After 48 h, cells were collected and centrifuged at 800 rpm for 5 min. Cells were then washed with PBS twice and fixed in ice-cold 70% ethanol for 1 h, and treated with 100 µl of 50 mg/l propidium iodide for 30 min at 4°C in the dark. Data were analyzed by CELL Quest software (BD Biosciences, San Jose, CA, USA). Apoptosis was examined using acridine orange/ethidium bromide (AO/EB) fluorescence staining. Transfectants (1×10^5^ cells) were replated in six-well plates. After 24 hours, cells were washed three times with phosphate-buffered saline (PBS) then stained with AO/EB for 5 min and visualized immediately under a fluorescence microscope (LEICA CTR4000B). The percentage of apoptotic cells was then calculated by the formula: percentage of apoptotic cell (%)  =  (amount of apoptotic cell/total cell examined) ×100%.

### Western blot analysis

Forty-eight hours after transfection, cells were harvested and lysed in M-PER Mammalian Protein Extraction Reagent (Pierce, Thermo Scientific, Cramlington, UK) containing a protease inhibitor cocktail (Sigma Aldrich, St. Louis, MO). A total of 50 µg of protein lysates were separated by sodium dodecyl sulphate/polyacrylamide gel electrophoresis (SDS-PAGE) and then transferred onto a PVDF membrane (Bio-Rad, Hercules, CA, USA). The primary antibodies were used: c-Jun (#9165, cell signaling Technology, Danvers, MA), p-c-Jun (#2361, cell signaling),cleaved caspase-3 (#9661, Cell Signaling), p53 (sc-126, Santa Cruz, CA), BAX (#9942, cell signaling), and GAPDH (Southern Biotech,Birmingham, USA) was used as a control. Proteins were visualized using an enhanced chemiluminescence kit (Amersham Pharmacia Biotech, Piscataway, NJ, USA).

### Statistical analysis

Statistical analyses were performed with SPSS software (version 16). Student's *t*-test, the χ2 test, and Fisher's exact test were used to compare methylation status and clinicopathological parameters. For all tests, *p*<0.05 was considered statistical significance.

## Results

### ZNF545 is downregulated in breast tumors and cell lines

**Figure 1:**
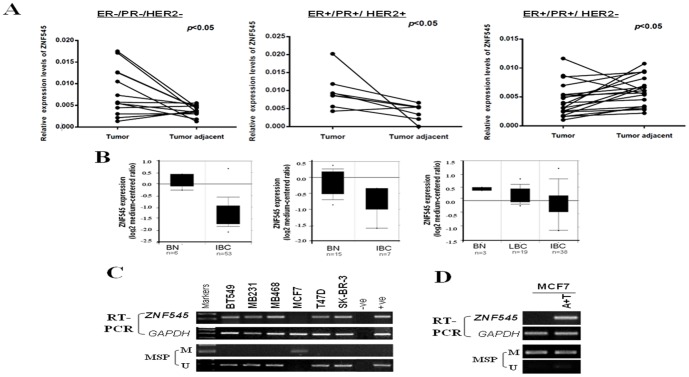
Downregulation of ZNF545 in breast cancer. (A) ZNF545 expression in primary breast tumor tissues and paired surgical margin tissues were evaluated using quantitative RT-PCR analysis. (B) Reduced expression of ZNF545 in breast cancer. Data extracted from cancer microdatabase Oncomine: https://www.oncomine.org/. BN: Normal Breast tissues; IBC: Invasive Breast Carcinoma; LBC: lobular breast carcinoma. (C) ZNF545 expression by semi-quantitative RT-PCR and methylation status of the ZNF545 promoter by MSP in breast cancer cell lines. GAPDH was used as a control. (D) Pharmacological demethylation restores expression of ZNF545 in MCF7 cell treated with Aza combined with TSA (A+T), accompanied by demethylation of the promoter. M, methylated; U, unmethylated.

We firstly examined ZNF545 expression in paired breast tumor tissues with different ER/PR/HER2 status by quantitative real-time PCR (qRT-PCR). We found that expression of ZNF545 was downregulated in 89.5% (17/19) of Luminal A (ER+/PR+/HER2−) subtypes, but increased in 69.2% (9/13) of triple negative (ER−/PR−/HER2−) and 87.5%(7/8) of Luminal B (ER+/PR+/HER2+) breast cancer tissues, compared with their adjacent non-tumor tissues (Fig. 1A, Table 2). We also detected ZNF545 expression in six breast cancer cell lines by semi-quantitative RT-PCR. Results showed that ZNF545 expression was silenced in MCF7 cells (Fig. 1C). Moreover, ZNF545 was significantly downregulated in breast cancer (Fig. 1B, Table 3), through analyzing the online microarray database (Oncomine, Compendia Bioscience, Ann Arbor, MI). These results suggest that ZNF545 is a candidate tumor suppressor for the Luminal A subtype breast cancer.

**Table 2:** Relationship between ER/PR/HER2 status and ZNF545 expression in paired breast cancer tissues.

Variable	No.paired cases	Expression rate	p-value
**ER+, PR+, HER2+**			0.0147
Downregulation	1	12.5%	
Upregulation	7	87.5%	
**ER+, PR+, HER2−**			0.0419
Downregulation	17	89.5%	
Upregulation	2	10.5%	
**ER−, PR−, HER2−**			0.0252
Downregulation	4	30.8%	
Upregulation	9	69.2%	

**Table 3:** Reduced expression of ZNF545 in breast cancer.

Dataset	Tissue type	Sample number	Median of expression intensity (log 2)	Fold change	*p*-value (normal vs cancer)
TCGA Breast	Breast	61	0.463		
	ICBC	3	−0.368	−1.835	0.002
	MBC	3	0.064	−1.259	0.007
	IC	76	0.150	−1.264	1.14E-05
	IDC	389	0.167	−1.329	2.87E-09
	ILC	36	0.275	−1.230	0.014
Zhao breast	Breast	3	0.480		
	LC	19	−0.180	−1.482	2.30E-04
	IDC	38	0.145	−1.196	3.49E-04
Karnoub breast	Breast	15	−0.385		
	ILC	7	−0.797	−1.475	0.006
Finak breast	Breast	6	−0.105		
	IC	53	−2.768	−5.909	6.26E-13

Data extracted from cancer microdatabase Oncomine: https://www.oncomine.org/. ICBC: Intraductal Cribriform Breast Adenocarcinoma; MBC: Male Breast Carcinoma; IC: Invasive Breast Carcinoma; IDC: invasive ductal breast carcinoma; ILC: invasive lobular breast carcinoma; LC: lobular breast carcinoma.

### Promoter methylation mediates ZNF545 downregulation in breast cancer

Abnormal promoter methylation is an important mechanism for TSG silencing in carcinogenesis [24], [25]. To determine whether promoter methylation leads to ZNF545 silencing, we detected ZNF545 methylation in breast tumor cell lines by MSP. We found that ZNF545 was only methylated in MCF7 cell line, consistent with its silencing. We further treated MCF7 with Aza and TSA, and found that ZNF545 expression was restored along with increased unmethylated promoter alleles (Fig. 1C).

**Figure 2:**
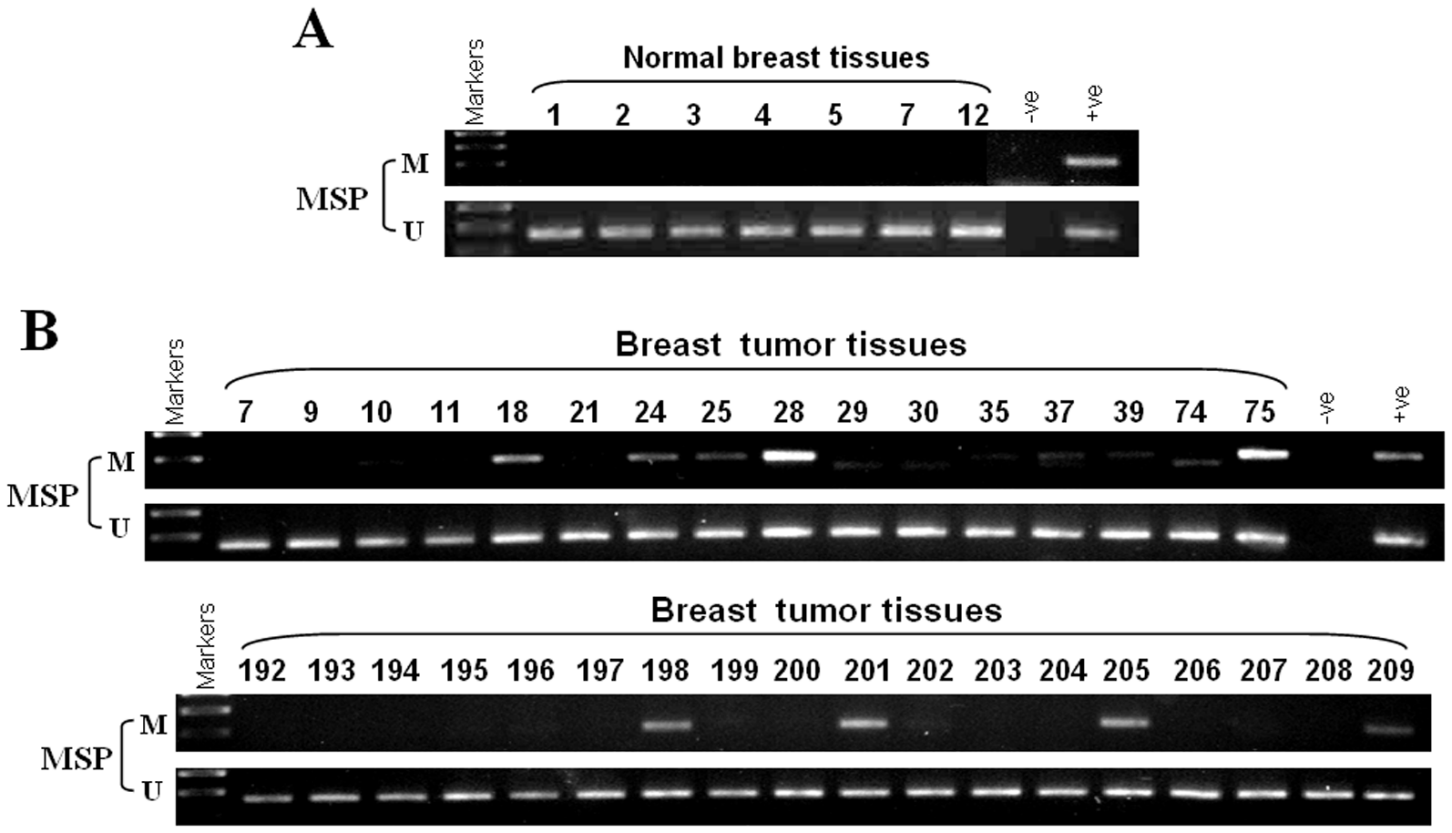
*ZNF545* is frequently methylated in primary breast tumors. (A) Methylation of *ZNF545* by MSP in normal breast tissues. (B) Representative analysis of methylation of the *ZNF545* promoter in breast tumor tissues. M, methylated; U, unmethylated.

To investigate ZNF545 promoter methylation in breast tumor tissues, MSP was used to examine 128 primary breast carcinomas tissues and seven normal breast tissues. ZNF545 methylation was detected in 37 out of 128 (29%) breast cancer tissue, but not in normal breast tissues (Fig. 2, Table 4), implying that methylation-mediated ZNF545 inactivation is a common event in breast cancer. These results suggest that ZNF545 is under tumor-specific methylation in breast cancer.

**Table 4:** Promoter methylation status of ZNF545 in primary breast tumors.

Tissue	Samples number	*ZNF545* promoter methylated status		Frequency of methylation
		methylated	unmethylated	
Breast tumor	128	37	91	29%
Normal breast	7	0	7	0

We further analyzed the relationship of ZNF545 methylation with clinicopathological features of breast cancer patients, including age, tumor grade, tumor size, lymph node metastasis, ER status, PR status and HER2 status, but no significant correlation was observed (Table 1).

### ZNF545 inhibits breast tumor cell clonogenicity and proliferation

**Figure 3:**
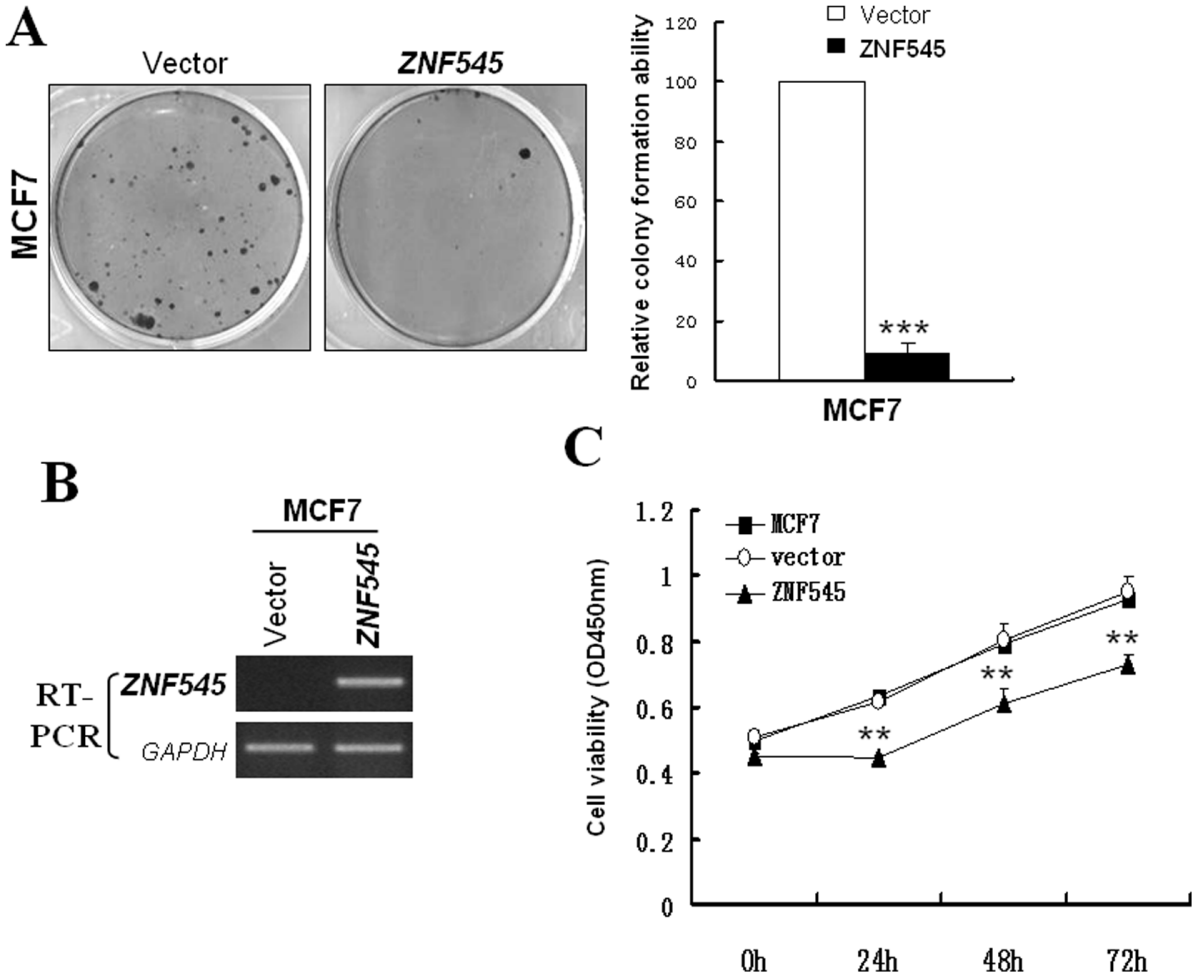
Effects of *ZNF545* on colony formation and cell proliferation of MCF7cells. (A) Representative colony formation assay and Quantitative analysis of colony formation. The numbers of G418-resistant colonies in vector-transfected controls were set to 100%, Values are expressed as the mean±SD from three experiments, and the asterisk indicates the statistical significance compared to the controls (***, p<0.001). (B) Expression of ZNF545 by RT-PCR in vector- and ZNF545-transfected MCF-7 cells. (C) CCK-8 assay for cell proliferation on vector- and ZNF545-transfecetd MCF7 cells. Asterisks indicate a significant level of proliferation compared with controls (**, p<0.01).

To further determine whether ZNF545 is a functional TSG in breast cancer, the effect of ZNF545 on MCF7 cell proliferation was examined by colony formation assay and CCK8 assay. Results showed that ZNF545 markedly reduced the efficiency of MCF7 colony formation to ∼10% compared to controls (***p<0.001) (Fig. 3A). RT-PCR confirmed ZNF545 expression in ZNF545-transfected MCF cells (Fig. 3B). After transfection with ZNF545 in MCF7 cells, cell viability significantly decreased at 24 h, 48 h and 72 h (**p<0.01) (Fig. 3C).

### ZNF545 induces cell cycle arrest and apoptosis of MCF7 cells

**Figure 4:**
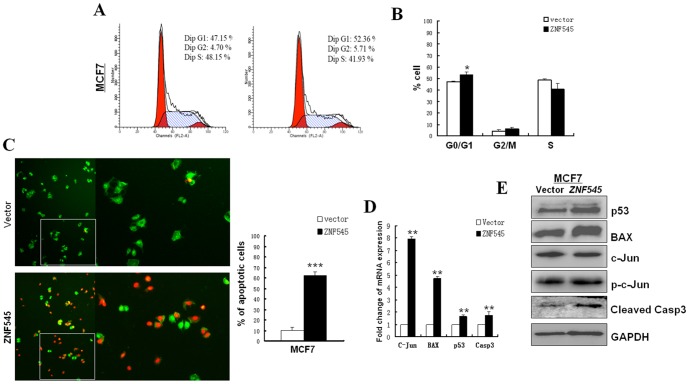
Ectopic expression of ZNF545 induced apoptosis and cell cycle arrest in MCF7 cells. (A) Anlysis of cell cycle distribution of vector-, ZNF545-transfected MCF7 cells. The distribution and percentage of cells in G1, S and G2/M phase of the cell cycle are indicated. B) Representative flow cytometry histograms of cell cycle alterations. The assay was performed in triplicate (*, *P*<0.05).(C), Apoptosis was examined using Acridine orange/ethidium bromide (AO/EB) fluorescence staining. The percentage of apoptotic cells is indicated(***, *P*<0.001). (D). Expression levels of C-Jun, BAX, p53 and Casp3 were evaluated by real-time PCR in vector-, ZNF545-transfected MCF7 cells. (E). Expression levels of C-Jun, BAX, p53 and Casp3 were evaluated by westernblot in vector-, ZNF545-transfected MCF7 cells.

Flow cytometric analysis of cell cycle and AO/EB staining were used to assess the mechanism of ZNF545 in inhibiting cell proliferation. ZNF545 obviously increased the number of MCF7 cells in the G0-G1 phase from 47.42% to 52.36%(*p<0.05) compared to controls (Fig. 4A, B). These results suggested that cell proliferation inhibition by ZNF545 is likely mediated through cell cycle arrest at G0/G1.

AO/EB staining is used to detect early apoptotic cells (stained green with yellowish dots as well as nuclear fragmentation and blebbing of cytoplasm) and late apoptotic cells (stained orange with condensed and often fragmented nuclei). AO/EB staining showed uniformly green control cells with normal morphology, whereas green early apoptotic cells with chromatin condensation occurred in ZNF545-expressing MCF7 cells, with orange later apoptotic cells with fragmented chromatin and apoptotic bodies also appearing (Fig. 4C). These results suggest that ZNF545 could also induce apoptosis in MCF7 cells.

### ZNF545 increased the expression of c-Jun/AP1, BAX, p53 and Caspase 3

We also examined its potential downstream target genes in ZNF545-expressing MCF7 cells by qRT-PCR, and found that ZNF545 could upregulate expression of c-Jun/AP1, BAX, p53 and Caspase 3 in mRNA level and protein level (Fig. 4D,4E). Thus ZNF545 may act as a transcript factor in breast cancer.

## Discussion

ZNF545 is reportedly a functional tumor suppressor in multiple cancers and is silenced by promoter methylation by our group [14], [26]. This is the first study to elucidate the direct relationship between ZNF545 and breast cancer. In this study, ZNF545 was silenced by promoter methylation in breast tumor cells. Restoring ZNF545 expression through demethylation treatment with Aza and TSA in MCF7 cells indicated that promoter methylation was the primary mechanism for its silencing. As ZNF545 was downregulated in breast cancer with ER+, PR+ and HER2-, compared with paired adjacent non-tumor tissues, ZNF545 may function as a candidate tumor suppressor in Luminal A subtype breast cancer. Tumor suppressive function of ZNF545 was thus studied in Luminal A subtype breast tumor cells. Ectopic expression of ZNF545 in MCF7 cells remarkably suppressed clonogenicity, inhibited cell proliferation and induced apoptosis, suggesting that ZNF545 is a tumor suppressor in breast cancer.

DNA methylation is a pivotal regulatory mechanism in gene transcription [27], and aberrant DNA methylation mediates TSG silencing in carcinogenesis [2]. Our previously studies have demonstrated that ZNF545 is frequently silenced by promoter methylation in multiple carcinomas including esophageal, nasopharyngeal, gastric, colorectal, and breast cancer [14], [26], [28]. To identify the relationship between the clinicopathological features and methylation status of ZNF545 in breast tumorigenesis, and to develop biomarkers for breast cancer detection and prognosis, ZNF545 methylation was examined in 29% of primary breast tumor tissues, but not in normal tissues, consistent with our previous study [14]. No significant associations were found between clinicopathological features and methylation status, although this needs to be further confirmed by studies using more samples.

ZNF545 is a novel member of the KRAB-containing zinc finger protein (KRAB-ZFP) family identified [29]. KRAB exerts its unique nuclear localization activity by interacting with its corepressor KAP1 (kinesin II-associated protein), and functions as a transcriptional repressor [30]. ZFP TF represses gene transcription by binding to target promoters. Thus, revealing the target molecules regulated by ZNF545 will be crucial for understanding the molecular basis of its tumor suppressive effect. Both NF-κB and AP-1 signaling pathways play important roles in the regulation of cell proliferation, survival, apoptosis, and malignant transformation [31], [32]. Previous reports revealed that the ectopic expression of ZNF545 suppresses AP-1 and NF-κB signaling, and inhibits the expression of multiple oncogenes in tumor cells [14]. We found that ZNF545 increased the transcription of c-Jun/AP1, BAX, p53 and Caspase 3, and then confirmed by Western blot, thus may serve as a transcriptional repressor in Luminal A subtype breast cancer. However, a systematic approach (gene expression array or RNASeq) to study the role of ZFP545 would be much more beneficial and may reveal additional targets.

In summary, this report is the first to show that ZNF545 is a functional TSG in breast cancer, through inhibiting cell growth and inducing apoptosis, and its tumor-specific methylation may serve as a potential tumor marker for breast cancer.
